# Comparison between spectral analysis and symbolic dynamics for heart rate variability analysis in the rat

**DOI:** 10.1038/s41598-017-08888-w

**Published:** 2017-08-16

**Authors:** Luiz Eduardo Virgilio Silva, Victor Rezende Geraldini, Bianca Potratz de Oliveira, Carlos Alberto Aguiar Silva, Alberto Porta, Rubens Fazan

**Affiliations:** 1 Department of Physiology, Ribeirão Preto Medical School, University of São Paulo, Ribeirão Preto, SP Brazil; 2 Department of Computer Science, Institute of Mathematics and Computer Science, University of São Paulo, São Carlos, SP Brazil; 3 Department of Biomedical Sciences for Health, University of Milan, Milan, Italy; 4 Department of Cardiothoracic, Vascular Anesthesia and Intensive Care, IRCCS Policlinico San Donato, San Donato Milanese, Milan, Italy

## Abstract

Spectral analysis of heart rate (HR) has been widely used to assess the autonomic cardiovascular control. A nonlinear approach, known as symbolic analysis, has been reported to be very useful to assess the autonomic control of cardiovascular system in humans, but very few studies reported on the differences between these two approaches on experimental models. Two distinct approaches were used to elicit autonomic changes in conscious Wistar rats: (1) pharmacological blockade of cardiac autonomic receptors with atenolol (ATE, N = 9) or methylatropine (ATR, N = 9) and (2) mild changes in arterial pressure (AP) induced by phenylephrine (PHE, N = 9) or sodium nitroprusside (NPS, N = 9). Series of cardiac interval (CI) and systolic AP (SAP) were assessed using spectral analysis and symbolic dynamics. Results show that, for spectral analysis, the power in high frequency band of CI and the power in low frequency band of SAP are the most reliable indices of vagal and sympathetic modulation, respectively. For symbolic analysis, results point 0V% and 1V% to be related to sympathetic and 2UV% to vagal modulation. Interestingly, the incidence of 1V patterns, hitherto with unknown meaning, was revealed the best index of sympathetic modulation in the rat and should be accounted for in the future studies.

## Introduction

The knowledge of the link between oscillatory components of heart rate (HR) and autonomic modulation of the heart, as demonstrated by Akselrod in early eighties^1^, has opened a fruitful field of investigation. While the HR oscillations at high frequency, coupled to respiratory rhythm, would reflect the parasympathetic cardiac modulation, slower oscillations (low frequencies) would be associated to the sympathetic control^2^.

While many studies reported data in agreement to the previous assumptions, several others researches raised contradictory findings^3^. The main source of discussion concerns to the meaning of the low frequency (LF) oscillatory components. Most data indicate that oscillations of HR in LF represent a combination of both sympathetic and parasympathetic modulation of the autonomic control to the heart^4^.

One of the main drawbacks of spectral analysis is that it is a linear method. In other words, it considers HR variability (HRV) time series as composed by a linear combination of the independent oscillatory components, where interactions between those components are neglected. A nonlinear alternative approach to spectral analysis was proposed by Porta *et al*., namely symbolic analysis^5^. The mathematics behind symbolic dynamics is quite different from spectral analysis, where no linearity is assumed and small sequences of values are assessed instead of long sinusoids.

Several studies showed that some indexes provided by symbolic dynamics are highly correlated with the sympathetic and parasympathetic modulation to the heart and vessels^6^. In addition, studies comparing these approaches provided solid evidences of the superiority of symbolic over spectral analysis^6–10^. Nevertheless, most of the studies are in humans and there is a lack about the validity of the symbolic analysis of HRV is experimental models.

In the present study we compared spectral and symbolic analysis as tools to assess the autonomic cardiac modulation in conscious rats. Two different protocols were performed to elicit autonomic imbalance by distinct mechanisms. Spectral and symbolic analyzes were calculated from the same animals and the efficacy of both approaches to provide valid information about autonomic cardiac modulation were evaluated.

## Methods

Part of the data used in this study was obtained from recordings of a previously published study involving pharmacological cardiac autonomic blockade^11^. All experimental procedures adhered to the Guide for the Care and Use of Laboratory Animals prepared by the National Academy of Sciences and published by the National Institutes of Health (Copyright © 1996 by the National Academy of Sciences), and were approved by the Committee of Ethics in Animal Research of the School of Medicine of Ribeirão Preto, University of São Paulo, SP, Brazil (protocols n° 016/2013-1 and 193/2016). All recordings were performed in conscious animals.

### Cardiac autonomic receptors blockade

Male Wistar rats (N = 18, 280–300 g) were anesthetized with a ketamine/xylazine mixture (50/10 mg/kg, respectively) and instrumented with subcutaneous electrodes for electrocardiogram (ECG) recordings and a polyethylene catheter into the left femoral vein for drug infusion. Both ECG electrodes and the catheter were tunneled subcutaneously and exteriorized at the back of the neck.

Two days after the surgery, under continuous ECG recording, the animals were submitted to selective pharmacological blockade of cardiac autonomic receptors as follows: after basal recording (60 min) half of the animals (N = 9) received intravenously atenolol (ATE:4 mg/kg *in bolus* followed by 0.8 mg/kg/h of infusion) and half (N = 9) received methylatropine (ATR:2 mg/kg *in bolus* followed by 0.4 mg/kg/h of infusion) during one hour^11^.

### Mild changes in arterial pressure by phenylephrine and sodium nitroprusside

Male Wistar rats (N = 18, 280 − 300 g) were anesthetized with a ketamine/xylazine mixture (50/10 mg/kg, respectively) and instrumented with polyethylene catheters into the femoral artery and vein, for arterial pressure (AP) measurement and drug infusion, respectively.

Two days after the surgery, the arterial lines of conscious freely moving rats were connected to a pressure transducer (MLT844, ADInstruments, Bella Vista, Australia) attached to a Bridge Amplifier and a recording system (Power Lab 4/40, ADInstruments, Australia), while venous catheters were connected to a syringe infusion pump (BI-2008, AVS Projetos, Brazil). After basal (10 min) recordings, the AP of rats was raised (N = 9) or lowered (N = 9) by intravenous infusion of phenylephrine (PHE: 1 µg/50 µL/min) or sodium nitroprusside (NPS: 0.6 µg/10 µL/min). Changes in AP elicited reflex-mediated changes in sympatho-vagal balance as clearly seen by HR responses.

**Figure 1:**
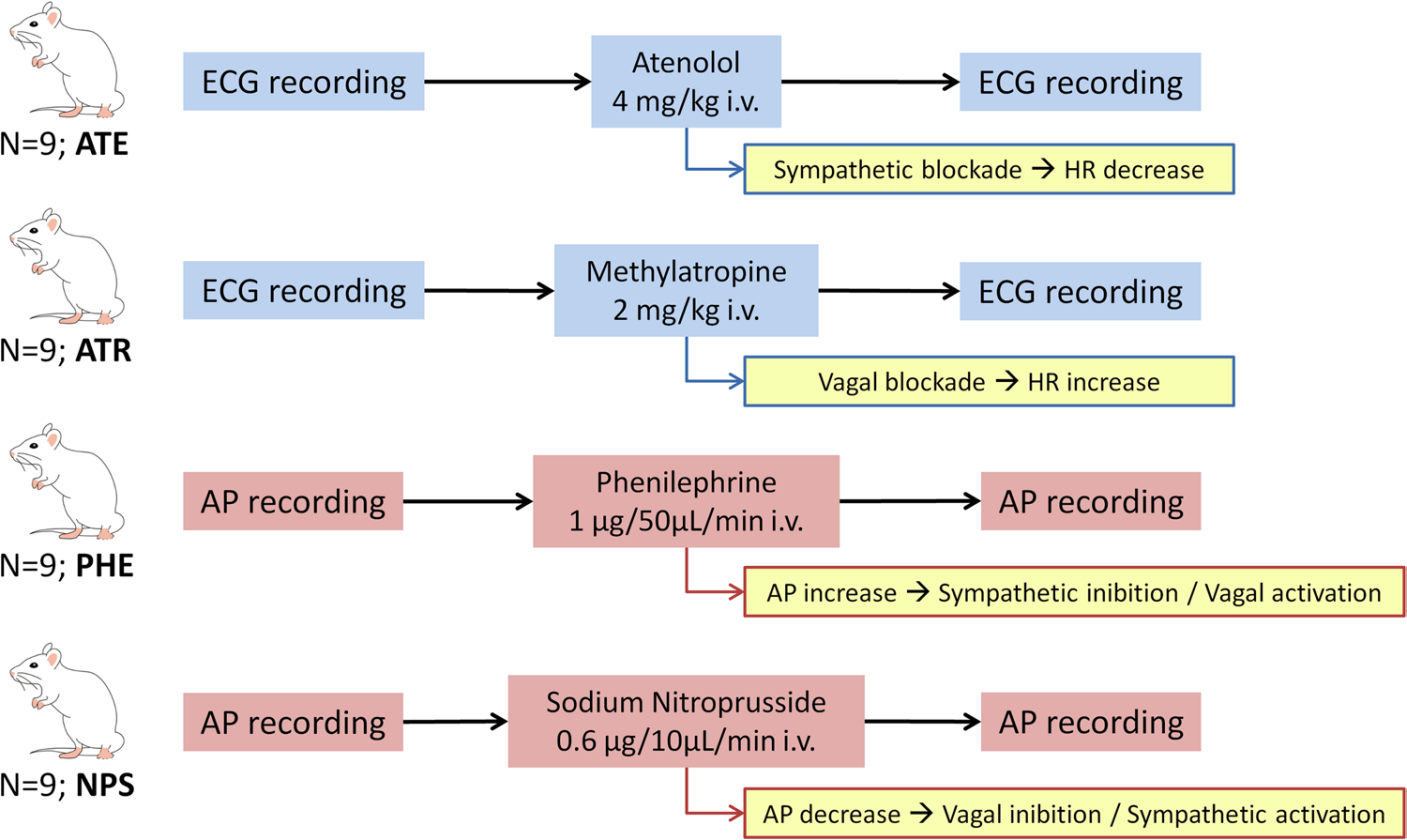
Schematic illustration of protocols and the hemodynamic and autonomic changes elicited at each group. ATE: atenolol group; ATR: atropine group; PHE: phenylephrine group; NPS: nitroprusside group; ECG: electrocardiogram; AP: arterial pressure; HR: heart rate.

Figure 1 illustrates the two protocols previously described, as well as hemodynamic and autonomic changes in ATE, ATR, PHE and NPS groups.

### Data processing

ECG or AP recordings were analyzed off-line by HRV or Blood Pressure Modules for LabChart computer software (ADInstruments, Castle Hill, NSW, Australia), respectively. Beat by beat series of RR or pulse intervals (both hereafter designated as cardiac interval, CI), as well as systolic AP (SAP) values, were calculated.

For symbolic dynamics analysis, artifacts were removed from beat by beat series of CI and systolic AP as follows: a moving average window of 50 points was used to calculate the series baseline. Next, values above and below inferior and superior thresholds set as the baseline plus and minus $p$ times the baseline (where 0.1 ≤ *p* ≤ 0.2) were removed. Removals do not exceed 1% of time series length.

For spectral analysis, the whole time series is divided into small segments and power spectrum is estimated into each segment (as described below). All segments were visually inspected by an expert and only those segments free of artifacts or transients were selected for analysis.

Mean values as well as the standard deviation of normal intervals (SDNN) and the root mean square of successive differences (RMSSD) were calculated from CI series^2^. Mean and standard deviation of SAP series was calculated. Each parameter was calculated in a sliding window (no overlap) of 1500 beats, taking the average as the final value.

### Spectral Analysis

Power spectral densities (PSD) of CI and SAP series were estimated by the fast Fourier transform (FFT), using Welch’s protocol with Hanning window^12^. First, series were interpolated at 10 Hz by cubic spline method in order to make time evenly spaced (a precondition of Fourier transform). Next, interpolated series were divided into half-overlapping segments of 512 samples. Following, after Hanning windowing, each segment had its spectrum estimated by the periodogram. The windowing is intended to attenuate side effects, which led to spectral leakage^13^. PSD was integrated in very low (VLF: 0 to 0.2 Hz)-, low (LF: 0.20 to 0.75 Hz)- and high-frequency (HF: 0.75 to 3.0 Hz) bands^14, 15^. The final result is the average of all selected segments. Powers of integrated bands are expressed in absolute (ms^2^) and normalized (nu) units. The LF/HF ratio was also calculated for assessment of the sympathovagal balance^14–17^.

### Symbolic Dynamics

The symbolic dynamics method, proposed by Porta^5^, aims to convert the CI and SAP series in a sequence of symbols and evaluates the dynamics of each three consecutive symbols (words). First, a procedure known as uniform quantization is applied to the CI or SAP series, where the full range of values is divided into six equal levels. Each quantization level is represented by a symbol (0 to 5) and all points within the same level will be assigned the same symbol. Next, sequences of three consecutive symbols (words) are evaluated and classified according to its variation pattern: zero variation (0V), one variation (1V), two like variations (2LV) or two unlike variations (2UV).

The 0V family comprises words where there is no variation between symbols, i.e., all symbols are equal. The sequences {0,0,0} and {3,3,3} are examples of sequences from this class. The 1V family represents words that have only one variation from one symbol to another, i.e. sequences with two consecutive equal symbols and one different. Examples of sequences of this family are {5,2,2} and {0,0,1}. The 2LV family is composed of words containing three different symbols but with the same variations direction, i.e. in ascending or descending order. Examples of sequences of this family are {1,2,5} and {3,2,1}. Lastly, 2UV family comprises sequences that form a peak or a valley, i.e. with two different variations, in opposite directions. The sequences {2,4,2} and {3,0,1} are examples of this family.

Once this classification is made for the entire series, the percentage of patterns classified in each family is used for analysis.

### Statistical Analysis

Data are presented as mean ± standard error of the mean. The *Shapiro-Wilk* test was applied to test the normality of data distribution. All comparisons were performed by paired *t-*test or *Wilcoxon* rank test when required. The significance level was set at P < 0.05.

### Data Availability

The datasets generated and analysed during the current study are available from the corresponding author on reasonable request.

## Results

While sympathetic receptor blockade with ATE caused a small, but significant, bradycardia, a marked rise in HR was elicited by parasympathetic blockade with ATR. Moreover, a remarkable reduction of HRV, expressed by both SDNN and RMSSD, were seen after either ATE or ATR (Table 1).

**Table 1:** Hemodynamic and time domain HRV parameters obtained from the groups of rats injected with atenolol (ATE) and methylatropine (ATR).

	Basal_ATE_	ATE	Basal_ATR_	ATR
Mean RR (ms)	165.8 ± 3.2	175.0 ± 4.3*	153.2 ± 2.7	130.3 ± 3.1*
SDNN (ms)	7.1 ± 0.2	3.8 ± 0.6*	6.1 ± 0.4	1.9 ± 0.2*
RMSSD (ms)	4.2 ± 0.3	2.9 ± 0.5*	3.7 ± 0.2	0.9 ± 0.1*

Basal_ATE_: basal period prior to atenolol infusion; Basal_ATR_: basal period prior to methylatropine infusion; RR: RR interval; SDNN: standard deviation of normal intervals; RMSSD: root mean square of successive differences; *different from respective basal. P < 0.05.

Intravenous infusion of PHE or NPS caused, as expected, increase and reduction of AP, respectively. Changes in pressure led to reflex changes in HR at the opposite direction. HRV (SDNN and RMSSD) lowered during NPS infusion but increased during PHE infusion. On the other hand, standard deviation of AP did not change during either NPS or PHE infusion (Table 2).

**Table 2:** Hemodynamic and time domain variability parameters obtained from the groups of rats injected with sodium nitroprusside (NPS) and phenylephrine (PHE).

	Basal_NPS_	NPS	Basal_PHE_	PHE
Mean PI (ms)	181.2 ± 4.4	159.2 ± 3.9*	166.6 ± 3.7	190.3 ± 3.9*
SDNN (ms)	7.1 ± 0.5	5.2 ± 0.5*	4.6 ± 0.6	8.2 ± 1.0*
RMSSD (ms)	6.3 ± 0.6	4.6 ± 0.4*	3.7 ± 0.4	9.4 ± 1.2*
Mean SAP (mmHg)	120.6 ± 2.0	106.0 ± 1.7*	118.6 ± 3.3	151.3 ± 5.3*
SD SAP (mmHg)	2.93 ± 0.24	3.26 ± 0.24	2.81 ± 0.32	3.48 ± 0.35

Basal_NPS_: basal period prior to NPS infusion; Basal_PHE_: basal period prior to PHE infusion; PI: pulse interval; SAP: systolic arterial pressure; SDNN: standard deviation of normal intervals; RMSSD: root mean square of successive differences; SD: standard deviation; *different from respective basal. P < 0.05.

**Figure 2:**
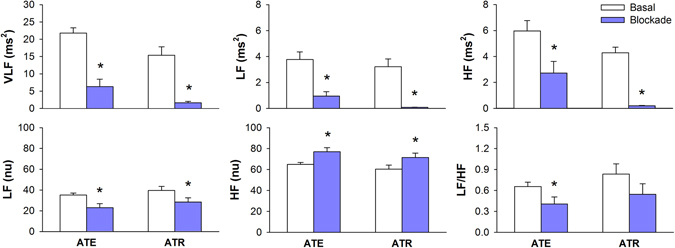
Spectral analysis of RR series during basal period and after cardiac autonomic blockade with atenolol (ATE) or methylatropine (ATR). VLF: power at very low frequency band; LF: power at low frequency band; HF: power at high frequency band; nu: normalized units; *different from respective basal. P < 0.05.

Figure 2 shows the power of CI spectra before and after autonomic receptor blockades (ATE or ATR) at VLF, LF and HF bands. In line with the decrease in overall HRV (lower SDNN) induced by either ATE or ATR, power of CI spectra expressed in absolute units (ms^2^) decreased after the infusion of both blockers at the three frequency bands studied. Either ATE or ATR led to a decrease in the power of CI spectra in LF and an increase in HF band when expressed in normalized units (nu). LF/HF ratio decreased after ATE but not after ATR infusion.

**Figure 3:**
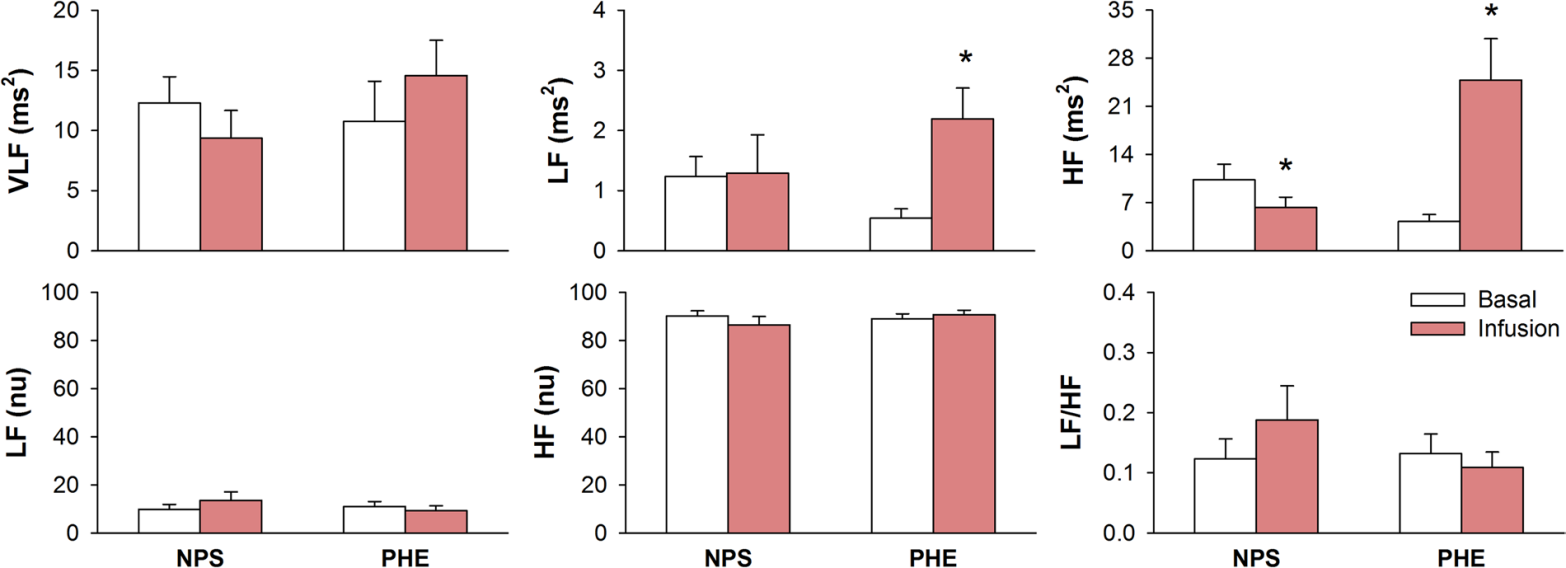
Spectral analysis of pulse interval series during basal period and after infusion of sodium nitroprusside (NPS) or phenylephrine (PHE). VLF: power at very low frequency band; LF: power at low frequency band; HF: power at high frequency band; nu: normalized units; *different from respective basal. P < 0.05.

Results of spectral analysis of CI during sympathetic or parasympathetic activation (changes in AP by NPS or PHE, respectively) are shown in Fig. 3. VLF power of CI spectra was not affected by NPS or PHE. Vagal activation induced by the rise in AP (PHE) led to a marked increase in the power of CI spectra at both LF and HF bands. NPS reduced the power of CI spectra at HF but did not affect LF band. When expressed in normalized units (or LF/HF), changes in AP did not affect CI spectra.

**Figure 4:**
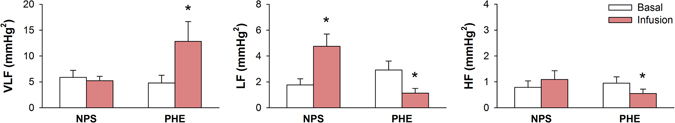
Spectral analysis of systolic arterial pressure variability during basal period and after infusion of sodium nitroprusside (NPS) or phenylephrine (PHE). VLF: power at very low frequency band; LF: power at low frequency band; HF: power at high frequency band; *different from respective basal. P < 0.05.

Figure 4 shows the power of SAP at the VLF, LF and HF before and after NPS or PHE. Rise in AP (PHE) was accompanied by an increase in the power of AP spectra at VLF but a decrease in pressure spectra at LF and HF bands. Sympathetic activation by the fall in AP (NPS) caused a marked increase in pressure spectra only at LF band.

**Figure 5:**
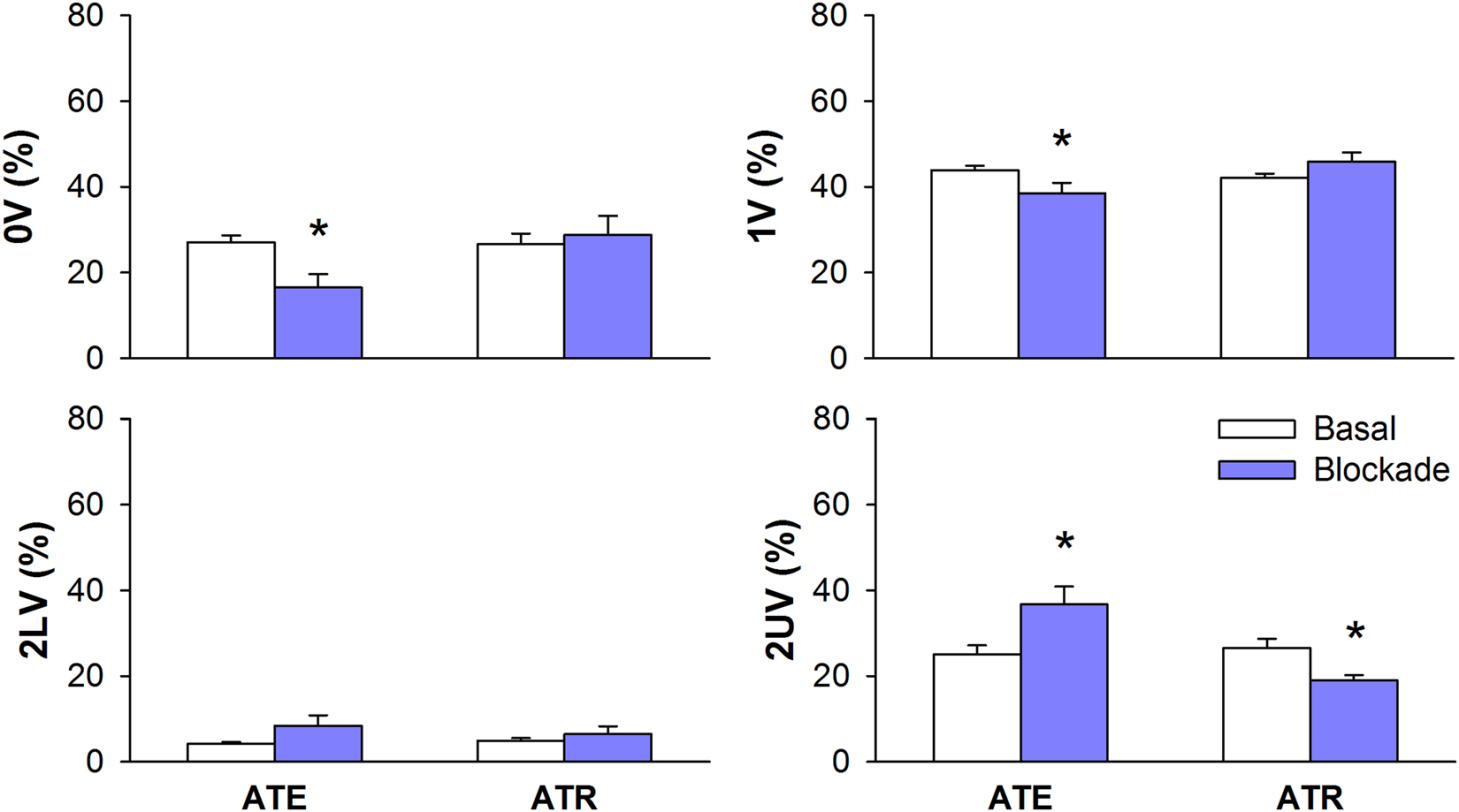
Symbolic dynamics analysis of RR series during basal period and after cardiac autonomic blockade with atenolol (ATE) or methylatropine (ATR). 0V: zero variation family; 1V: one variation family; 2LV: two like variations family; 2UV: two unlike variations family; *different from respective basal. P < 0.05.

Figure 5 shows the percentage of occurrence for each family of symbols (symbolic analysis) of CI, before and after autonomic receptors blockers ATE or ATR. Sympathetic blockade with ATE reduced the occurrence of 0V and 1V patterns whereas increased the incidence of 2UV. Parasympathetic blockade with ATR reduced the percentage of 2UV patterns. Also a trend of increase 1V% (P = 0.06) was seen after ATR infusion.

**Figure 6:**
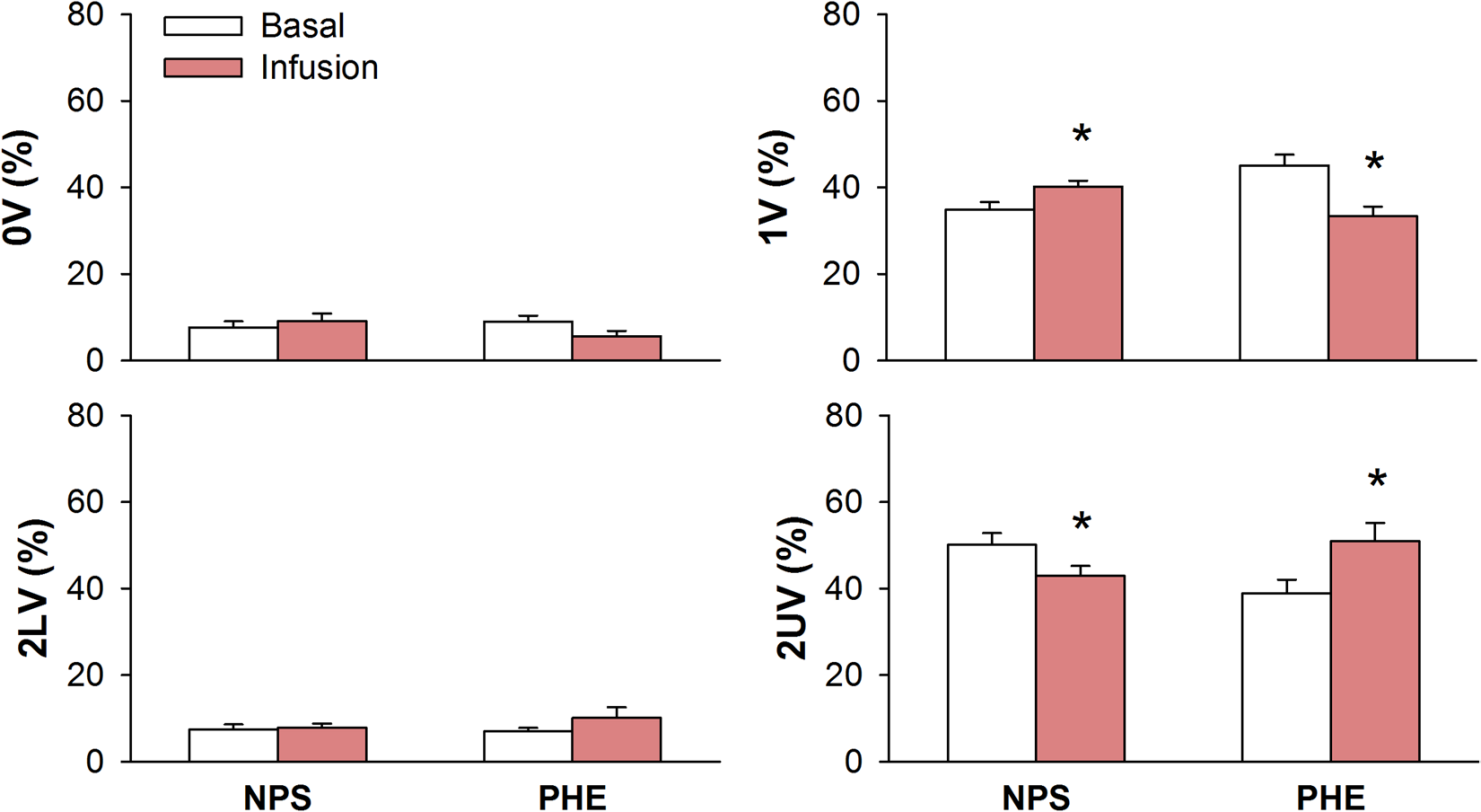
Symbolic dynamics analysis of pulse interval series during basal period and after infusion of sodium nitroprusside (NPS) or phenylephrine (PHE). 0V: zero variation family; 1V: one variation family; 2LV: two like variations family; 2UV: two unlike variations family; *different from respective basal. P < 0.05.

Symbolic dynamics of CI during pressure changes elicited by NPS or PHE are shown in Fig. 6. Sympathetic activation elicited by infusion of NPS increased the percentage of 1V patterns and reduced the occurrence of 2UV. On the other hand, PHE reduced the percentage of the pattern with 1V and increased the occurrence of 2UV.

**Figure 7:**
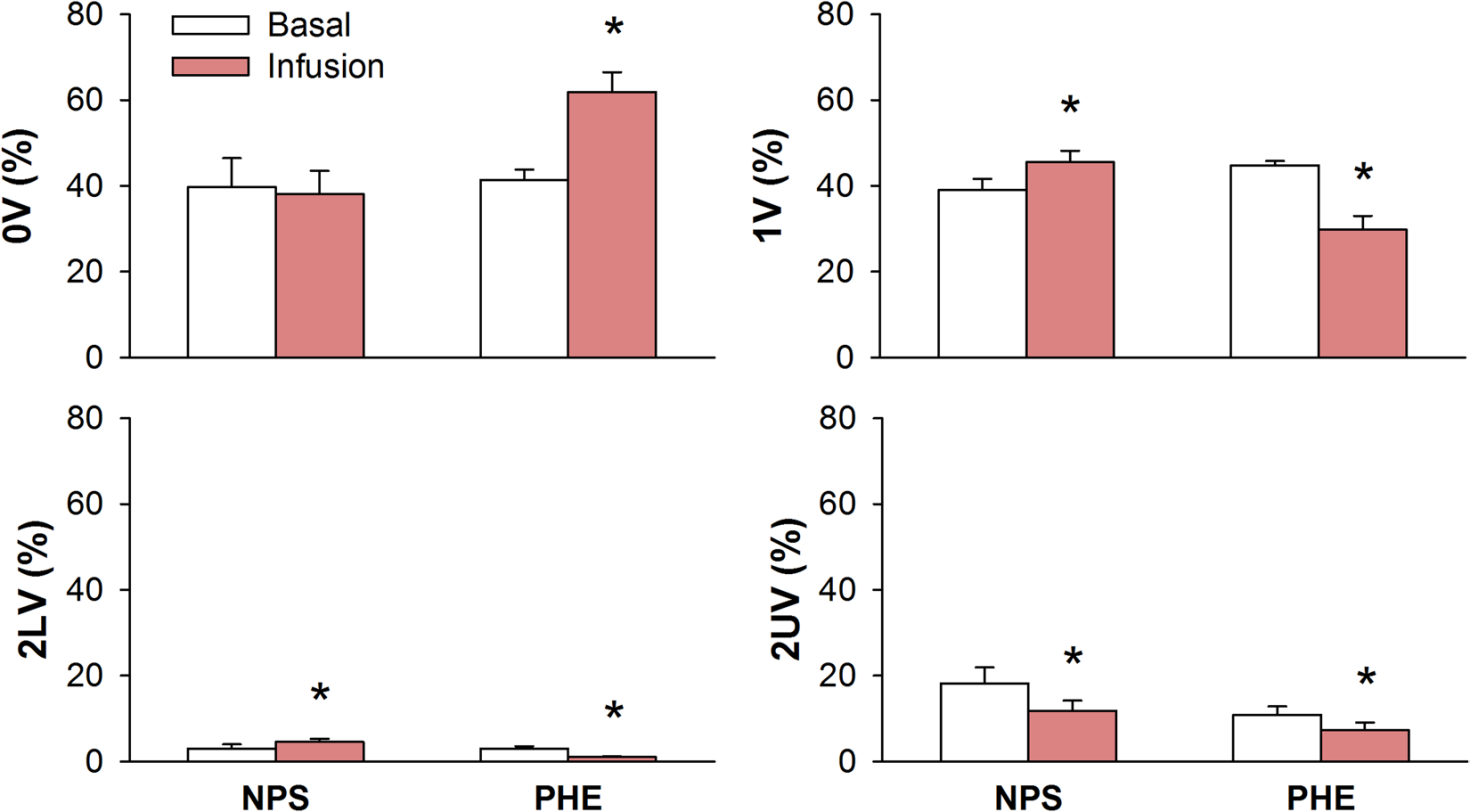
Symbolic dynamics analysis of systolic arterial pressure variability during basal period and after infusion of sodium nitroprusside (NPS) or phenylephrine (PHE). 0V: zero variation family; 1V: one variation family; 2LV: two like variations family; 2UV: two unlike variations family; *different from respective basal. P < 0.05.

Figure 7 shows the results of symbolic dynamics of SAP before and after infusion of NPS or PHE. Infusion of NPS increased 1V, 2LV and decreased 2UV percentages, whereas the infusion of PHE increased 0V but decreased 1 V, 2LV and 2UV percentages.

## Discussion

In this study, two distinct protocols were applied to elicit sympathovagal changes: (1) pharmacological blockade of cardiac autonomic receptors and (2) mild changes in AP causing baroreflex-mediated changes in autonomic drive. HRV under the blockade of adrenergic receptors by ATE and during rise in arterial pressure caused by PHE is mainly regulated by parasympathetic (vagal) control. In contrast, under muscarinic receptors blockade by ATR and during hypotension caused by NPS infusion, the variability of CI is mainly controlled by the sympathetic system. Moreover, in the protocols with NPS and PHE infusion, the autonomic imbalance is also expected to affect vessels. Therefore, during PHE infusion, the contribution of sympathetic modulation to SAP variability is attenuated, whereas during NPS infusion the contribution of sympathetic modulation to SAP variability is increased. Although the mechanisms used to promote changes in sympathovagal balance are distinct in the two protocols, the predominance of sympathetic or parasympathetic cardiac control must be detectable in both cases, if the analysis method is consistent.

For spectral analysis of HRV, the absolute power of CI spectra at HF band has been considered a strong indicator of parasympathetic cardiac modulation, whereas the LF power of CI in normalized units was identified more consistent than LF (abs) as an index of sympathetic modulation to the heart^6^. However, it is still a matter of debate and the LF band of CI spectra is considered by most researchers as composed by both sympathetic and parasympathetic influences^3, 4, 18^. Moreover, one has to consider that LF (nu), HF (nu) and LF/HF parameters are not independent, as one can be written as a function of the others^4^. For example, considering the power in normalized units, one can write: LF = 100-HF. Unless the sympathetic and parasympathetic modulation work in this very strict complementary interaction, the common association of LF (nu) to sympathetic modulation and HF (nu) to parasympathetic modulation is meaningless.

Results with HF power in absolute units were consistent for NPS, PHE and ATR protocols. For ATE, however, HF (abs) decreased when it was expected to increase. Results with power of LF band in normalized units were not consistent. It decreases for both ATE and ATR blockade whereas it did not change for NPS and PHE.

Spectral analysis of SAP variability showed that LF power is a robust index of sympathetic modulation to vessels. This might be due to the fact that the arterial system is poorly innervated by parasympathetic fibers, and thus, LF oscillations are not contaminated by sources other than sympathetic system as in the spectral analysis of CI^19^.

Several studies have reported the correlation of symbolic dynamics indexes with sympathetic and parasympathetic modulation to the heart and vessels, especially in humans^6, 8^. Those studies have shown that patterns of SAP and CI with 0V correlates to sympathetic modulation whereas CI sequences with 2UV (sometimes also 2LV) are linked to parasympathetic cardiac modulation. However, quite few studies have been conducted on experimental models^10^.

Here we showed that 2UV% of CI series is consistently altered in all protocols, representing a good index of the vagal modulation to the heart in rats. Against the odds, 0V% of CI is only significantly altered during ATE infusion. In addition, 0V% of SAP during infusion with PHE increased when it was expected to decrease. Therefore, 0V% seems not to be a robust index of sympathetic modulation for CI and SAP in rats.

On the other hand, one of the most interesting findings of this study concerns to the percentage of 1V patterns. Essentially, it represents sequences of three symbols where two consecutive symbols are equal and the remaining is different. This type of pattern is very similar to patterns belonging to 0V family, except for one symbol. Results suggest that 1V% as a very consistent index of sympathetic modulation, for both CI and SAP variability. Therefore, for rats, 1V patterns may better represent sympathetic modulation than 0V patterns. The use of simulated data stressed the relevant association of 1V patterns with slower CI rhythmicities in rats^20^. Since symbolic dynamics has been studied, 1V patterns have been considered meaningless for HRV analysis. For rats, however, those patterns might carry important information about sympathetic modulation. This finding might be the consequence of the peculiar characteristic of the cardiac physiology and/or cardiovascular control in rats featuring faster heart rate and shorter temporal scales of vagal and sympathetic control compared to humans.

In a similar study with humans, the administration of ATR, PHE or NPS did not affect the power of HR spectra at LF or HF bands^8^. Nevertheless, indices from symbolic dynamics were consistently altered by these drugs. ATR caused 0V to increase and 2V (2LV + 2UV) to decrease. NPS increased 0V whereas PHE increased 2V. However, the percentage of 1V patterns did not change, reinforcing that the autonomic control does not affect those patterns in healthy humans^8^.

Tobaldini and cols^10^ have shown that, in experimental models of rats with congestive heart failure (CHF), the occurrence of both 0V and 1V are reduced, consistent to the lower sympathetic modulation observed in the CHF. It is known that CHF is followed by several disturbances in addition the autonomic imbalance, such as inflammation and impaired baroreflex^21, 22^. One could assume that those physiological alterations could produce oscillatory components in CI that possibly will be accounted for in the 0V family. Another possibility is that the sympathetic modulation could encompass both families 0V and 1V, depending on the situation, and therefore, these two families should be considered in the rat model. Of note, the same study showed that spectral analysis was not able to detect the autonomic changes elicited in rats by CHF^10^. The importance of the 1V pattern family was highlighted in humans as well, given that their rate of occurrences added independent prognostic information to a clinical model of CHF^23^.

In conclusion, symbolic dynamics seems to be more robust than spectral analysis to assess the autonomic modulation of rats. Even so, for spectral analysis, HF power of CI in absolute values was the most reliable measure of parasympathetic modulation and LF power of SAP was the most reliable measure of sympathetic modulation. For symbolic dynamics, the percentage of patterns in 1V family seems to be the best indicator of sympathetic modulation, whereas 2UV% was the best index of parasympathetic cardiac modulation. Therefore, 1V family seems to be an important source of information regarding sympathetic modulation and should be considered for the analysis of HRV in rats. Nevertheless, 0V family may still play some role in some situations and should not be discarded.
